# Accessory renal arteries in a Caribbean population: a computed tomography based study

**DOI:** 10.1186/2193-1801-2-443

**Published:** 2013-09-08

**Authors:** Peter B Johnson, Shamir O Cawich, Sundeep D Shah, William Aiken, Roy G McGregor, Hilary Brown, Michael T Gardner

**Affiliations:** Department of Surgery, Radiology, Anaesthetics and Intensive Care, Faculty of Medical Sciences, University of the West Indies, Mona Campus, Mona, Jamaica; Department of Clinical Surgical Sciences, Faculty of Medical Sciences, University of the West Indies, St. Augustine Campus, St. Augustine, Trinidad & Tobago; Department of Surgery, Cornwall Regional Hospital, Montego Bay, Jamaica; Section of Anatomy, Basic Medical Sciences, University of the West Indies, Mona Campus, Kingston 7, Mona, Jamaica

**Keywords:** Renal, Artery, Vasculature, Accessory, Variant

## Abstract

**Introduction:**

The commonest variation to the classic anatomic description of renal arterial supply is the presence of accessory renal arteries. The incidence varies widely according to ethnicity. There is no data on the prevalence of these anomalies in persons of Caribbean ethnicity.

**Methods:**

All CT scans done over two years from July 1, 2010 to June 30, 2012 were retrospectively evaluated. The anatomy of the renal arterial supply was reported from these studies and the anatomy of accessory renal arteries was documented.

**Results:**

There were 302 CT scans evaluated and accessory renal arteries were present in 109/302 (36.1%) CT scans, 95% confidence interval 30.6%, 41.4%. There were 71/309 (23.5%) patients with accessory arteries on the left and 54/309 (17.9%) had them on the right (p 0.087). Of these, 16 (14.7%) patients had bilateral accessory renal arteries present. The most common origin for the accessory arteries was the abdominal aorta in 108 (99.1%) cases and in 1 case the accessory artery arose from the coeliac trunk. There were 80 left sided accessory renal arteries: 17 (21.3%) upper polar and 27 (33.8%) lower polar arteries. Of 62 right sided accessory arteries, 14 (22.6%) were upper polar and 26 (42%) were lower polar arteries.

**Conclusion:**

This is the first population-based report of anatomic anomalies in renal arterial supply in a Caribbean population. These are important findings that may affect vascular and urologic procedures on persons of Caribbean ethnicity.

## Background

The classic description of renal arterial anatomy describes a single renal artery arising from the abdominal aorta and entering the renal hilum. It branches at or near the hilum into anterior and posterior branches and then progressively into lobar, inter-lobar and arcuate arteries, eventually supplying the parenchyma as end arteries(Anatomy 2005; Merklin & Michels 1958; Michels 1955). However, many patients have variations to this classic description (Harrison et al. 1978; Khamanarong et al. 2004; Sampaio & Passos 1992; Urban et al. 2001; Beregi et al. 1999; Ozkan et al. 2006). The most common variation is the presence of accessory renal arteries (Harrison et al. 1978; Khamanarong et al. 2004; Sampaio & Passos 1992; Urban et al. 2001; Beregi et al. 1999; Ozkan et al. 2006). The reported incidence ranges from 11.3% (Sungura 2012) to 59.5% (Budhiraja et al. 2013) of persons and varies widely with ethnicity (Ozkan et al. 2006;Sungura 2012; Budhiraja et al. 2013; Saldarriaga et al. 2008; Papaloucas et al. 2007; Santos-Soares et al. 2013).

Accessory arteries are clinically significant because they affect several operative procedures including graft deployment during endovascular aneurysm repair, open aneurysmorrhaphy, nephrectomy and renal transplantation. Therefore, it is important to have knowledge of anatomic variants in each population.

A literature review in Medscape, Medline, Pubmed, Embase and Scilo was performed on April 30, 2013 using the keywords: Caribbean, renal, artery, vascular, perfusion, anomaly and variants. This returned two case reports describing the presence of accessory renal arteries encountered during cadaveric dissections for anatomic teaching (Adekunle & Uche-Nwachi 2007; Rao et al. 2011). There were no reports of large population-based studies from the Caribbean. We carried out this retrospective study to evaluate the incidence and distribution of accessory renal arteries in unselected patients undergoing contrast enhanced computer tomography (CT) scanning of the abdomen at a regional referral centre in the northern Caribbean.

## Methods

This study was done at the University Hospital of the West Indies (UHWI) in Jamaica. This is a 500 bed hospital that serves as a major tertiary referral centre for Jamaica and a regional referral centre for northern Caribbean countries (Johnson et al. 2013). Therefore, the findings in this study should be representative of anatomic variations in the general population across the Anglophone Caribbean.

At the UHWI, multiphase CT and angiography (CTA) were introduced routinely in the radiology department in 2007 with the introduction of a Phillips Brilliance® 64 slice multi-detector CT scanner. Currently over 5000 CT scans are performed yearly at the UHWI. The institutional review board (University of the West Indies Faculty of Medical Science Ethics Committee) granted ethical approval to access these studies to evaluate the distribution of anatomic patterns. Written informed consent was obtained from the patient for the publication of this report and any accompanying images.

Two practicing radiologists independently evaluated images from all CT scan series of the abdomen and pelvis performed on a 64-slice CT scanner over a period of two years from July 1, 2010 to June 30, 2012. The anatomy of the renal arterial supply on each scan was reported from these studies. The images evaluated in this study included: all CTAs of the abdominal aorta and iliac arteries; all abdomino-pelvic CT scans with an arterial phase covering the abdominal aorta and iliac arteries; and all CT scans of the chest that adequately covered the entire abdomen in the arterial phase. We excluded the following studies: any CT scans done at referring facilities; any CT with incomplete demographic data; duplicated CT scans; CT scans without adequate arterial phases; and CT scans in patients who had prior nephrectomy as these patient would be expected to have disturbed anatomy. This study was performed to investigate renal arterial anatomy in patients with scans for a variety of indications. Therefore we could not reliably comment on the relationship of the ureters to any accessory arteries encountered.

Merklin and Michels 1958 proposed a classification for accessory arteries based on their origin and entry into the kidney. We utilized this classification to describe the variants encountered in our study. The main renal artery was considered to be a single dominant artery arising from the abdominal aorta that entered the renal hilum and branched into segmental arteries. Accessory renal arteries did not branch prior to entry into the renal parenchyma. They were classified based on the Merklin and Michels’ classification based on their origin from: (1) abdominal aorta; (2) main renal artery and (3) origin from other sources. We also classified the variations according to their entry into the kidney into: (1) upper polar; (2) hilar and (3) lower polar accessory arteries.

Data were extracted from the CT scans and recorded in a Microsoft Excel chart formatted with the above information. Descriptive statistics were generated using the SPSS statistical software and a statistical test of differences in proportions was used to compare the frequency or accessory arteries on the right and left sides.

## Results

There were 302 CT scans done over the study period that met the inclusion criteria. Accessory renal arteries were present in 109 (36.1%) patients, (95% confidence interval (CI): 30.6%, to 41.4%). There were accessory renal arteries present in 71/302 (23.5%) patients on the left and 54/302 (17.9%) patients on the right side. Sixteen (5.3%) patients had bilateral accessory renal arteries present. Although accessory renal arteries were commoner on the left, the difference did not attain statistical significance (23.5% vs 17.9%, p = 0.087).

According to the Merklin and Michels’ classification (Merklin & Michels 1958), the most common origin for accessory renal arteries was directly from the abdominal aorta in all cases. There was a single case in which the origin of the accessory renal artery came directly off the aorta antero-laterally at the same level as the coeliac trunk, coursed infero-laterally to the right to enter the upper pole of the right kidney. There were no cases in which the accessory artery took an origin from the main renal artery or any other vessel.

The anatomy was also described according to the entry point on the kidneys. There were 80 accessory renal arteries on the left side in 71 patients. Table 1 classifies these arteries according to their renal entry point. There were 62 accessory renal arteries on the right side in 54 patients and this is tabulated in Table 2.

**Table 1:** **Distribution of left-sided accessory renal arteries in a Caribbean population**

Entry point at kidney	Number of accessory renal arteries on the left side in 71 patients				
	1	2	3	4	Total
**Upper polar**	16	0	1	0	17
**Hilar**	28	6	2	0	36
**Lower polar**	21	4	1	1	27
					80

**Table 2:** **Distribution of right-sided accessory renal arteries in a Caribbean population**

Entry point at kidney	Number of accessory renal arteries on the right side in 54 patients				
	1	2	3	4	Total
**Upper polar**	13	0	0	1	14
**Hilar**	19	3	0	0	22
**Lower polar**	21	3	1	1	26
					62

## Discussion

In the developing human embryo the mesonephros, metanephros, adrenals and gonads are supplied by paired mesonephric arteries arising from the dorsal aorta (Hlaing et al. 2010; Felix 1911). The 3^rd^, 4^th^ and 5^th^ pairs of lateral mesonephric arteries supply the metanephros (Felix 1911). The caudal branches usually disappear, leaving a single persistent renal artery (Felix 1911). When more than one of these lateral mesonephric arteries persist, multiple accessory renal arteries result (Budhiraja et al. 2013; Hlaing et al. 2010; Felix 1911).

The presence of accessory renal arteries is one of the most common urogenital variants (Harrison et al. 1978; Khamanarong et al. 2004; Sampaio & Passos 1992; Urban et al. 2001; Beregi et al. 1999; Ozkan et al. 2006). It is well documented that the incidence of accessory renal arteries varies widely with ethnicity, ranging from 11.4% in Kenyans (Sungura 2012) to 59.5% in Indians (Budhiraja et al. 2013). Table 3 documents the variation in incidence according to ethnicity in published medical literature.

**Table 3:** **Incidence of accessory renal arteries in published literature according to ethnicity**

Author	Origin	Study population size	Accessory renal arteries	
			(n)	%
Sungura (2012)	Kenya	204 CT Angiograms	23	11.3%
Santos-Soares et al. (2013)	Brazil	50 cadaveric renal blocks	6	12.0%
Ozkan (2006)	Turkey	855 CT Angiograms	202	23.6%
Saldarriaga et al. (2008)	Colombia	390 cadaveric kidneys	98	25.1%
Papaloucas et al. (2007)	Greece	215 Angiograms	59	27.4%
Present study	Jamaica	302 Angiograms	109	36.1%
Budhiraja et al. (2013)	India	74 cadaveric kidneys	44	59.5%

Renal arterial anatomy was traditionally evaluated with catheter angiography but it has now been superseded by CT angiography (Urban et al. 2001; Sebastia et al. 2010; Das 2008; Smith et al. 1998) that reportedly can identify renal arterial anatomy with 66-100% sensitivity and 75-100% specificity (Smith et al. 1998; Sahani et al. 2005; Dachman et al. 1998; Pozniak et al. 1998). There is also the advantage of providing other information that may impact treatment such as demonstrating venous and collecting system anatomy that conventional angiography cannot offer (Sebastia et al. 2010).

Using CT angiography we have shown that the prevalence of accessory renal arteries is 36.1% in the Caribbean population. This incidence is greater than that seen in most other ethnicities (Table 2), exceeded only by the reported incidence in Northern India, where Budhiraja et al. (Budhiraja et al. 2013) reported an incidence of 59.5%. However, Budhiraja’s study only evaluated a small convenience sample of 37 cadavers used in anatomic teaching (Budhiraja et al. 2013). Most of the published reports in medical literature with more than 200 patients or cadavers (Ozkan et al. 2006; Sungura 2012; Budhiraja et al. 2013; Saldarriaga et al. 2008; Papaloucas et al. 2007; Santos-Soares et al. 2013) reported much lower incidence ranging from 11.2% in Kenya (Sungura 2012) to 27.4% in Greece(Papaloucas et al. 2007). When compared to these larger population-based studies (Ozkan et al. 2006; Sungura 2012; Budhiraja et al. 2013; Saldarriaga et al. 2008; Papaloucas et al. 2007; Santos-Soares et al. 2013), the incidence in persons of Caribbean ethnicity is higher than we expected. Similar to international data (Khamanarong et al. 2004; Sampaio & Passos 1992; Urban et al. 2001; Beregi et al. 1999; Ozkan et al. 2006; Sungura 2012; Budhiraja et al. 2013; Saldarriaga et al. 2008; Papaloucas et al. 2007; Santos-Soares et al. 2013), the accessory renal arteries were encountered more commonly on the left side (23.5% vs 17.9%) in our population.

Knowledge of the presence and distribution of accessory arteries is of paramount importance in renal transplantation. The presence of accessory arteries in a donor kidney is usually considered a contraindication to its use in transplant surgery (Sebastia et al. 2010). Since these are end arteries, the accessory arteries must be re-implanted and this would require several anastomoses and a prolonged ischemic time, leading to a theoretically higher incidence of renal failure, graft rejection and reduced graft function (Budhiraja et al. 2013; Sebastia et al. 2010; Das 2008).

Using Merklin and Michels’ classification (Merklin & Michels 1958), we evaluated the accessory origins. There were no cases of an accessory artery originating from the main renal artery. This was similar to the original findings published by Merklin and Michels (Merklin & Michels 1958), although other investigators have reported renal artery origins being present in 8% (Budhiraja et al. 2013) to 14% of cases (Talovic et al. 2007). In our series all of the accessories originated from the aorta. This was greater than the incidence reported in the literature where aortic takeoff is reported to occur in only 30.8% (Talovic et al. 2007) to 47.3% (Budhiraja et al. 2013).

The accessory origin may have significant impact on endovascular aneurysm repair because graft deployment and seating may need to be adjusted in the face of these anomalies. Even in open aneurysmorrhaphy, there must be careful planning of aortic cross clamp placement to prevent inadvertent occlusion of these end arteries and renal ischaemia (Schepens et al. 1994; Safi et al. 1996). In these cases, operative techniques would need to be modified and the informed consent process adjusted to reflect an increase in operative risk. For this reason, CT is well established as the primary modality to evaluate abdominal aortic aneurysms in terms of diagnosis and treatment planning (Errington et al. 1997; Simoni et al. 1996).

The entry point on the kidney is also important, especially with polar accessory arteries that enter the renal parenchyma directly outside of the hilum (Sebastia et al. 2010). In our series, 42% of the accessory renal arteries were lower polar arteries on the right side and 33.8% were lower polar arteries on the left side. Lower polar accessories tend to give important arterial supply to the upper ureter so any occlusion or inadvertent ligation could result in graft necrosis at the uretero-pelvic junction resulting in a leak or stenosis (Sebastia et al. 2010). With an aortic origin, they may also cause obstruction at the uretero-pelvic junction leading to impaired renal function (Shoja et al. 2008).

Upper polar arteries were less common, accounting for 22.6% (14/62) of the accessory arteries on present the right and 21.3% (17/80) of those on the left. They are still important because they are at risk for injury during dissection, especially those with an aortic origin that have a horizontal course (Sampaio & Passos 1992; Budhiraja et al. 2013).

## Conclusion

Approximately 36% of persons in a Caribbean population can be expected to have accessory renal arteries present. This is greater than the incidence reported in most large population-based studies in medical literature. The finding is commoner on the left side and in most cases they arise directly from the aorta. These are important findings that may affect vascular and urologic procedures on persons of Caribbean ethnicity.
